# Prophylactic protection from lethal henipavirus disease mediated by Nipah-derived defective interfering particles is influenced by challenge virus strain and viral species

**DOI:** 10.1016/j.ebiom.2025.105897

**Published:** 2025-08-25

**Authors:** Stephen R. Welch, Jessica R. Spengler, Jessica R. Harmon, JoAnn D. Coleman-McCray, Sarah C. Genzer, Katherine A. Davies, Teresa E. Sorvillo, Florine E.M. Scholte, Sergio E. Rodriguez, Joel M. Montgomery, Stuart T. Nichol, Christina F. Spiropoulou

**Affiliations:** a Viral Special Pathogens Branch, Division of High Consequence Pathogens and Pathology, Centers for Disease Control and Prevention, Atlanta, GA 30333, USA; b U.S. Department of Agriculture, Agricultural Research Service, Zoonotic and Emerging Disease Research Unit, National Bio and Agro-Defense Facility, Manhattan, KS 66506, USA; c CDC Foundation Assigned to Viral Special Pathogens Branch, Division of High Consequence Pathogens and Pathology, Centers for Disease Control and Prevention, Atlanta, GA 30333, USA

**Keywords:** Nipah virus, Hendra virus, Henipavirus, Viral zoonoses, Defective interfering viruses, Mesocricetus, Syrian hamsters, Models, Animal, Antiviral agents, Administration, Intranasal

## Abstract

**Background:**

Henipaviruses, including Nipah and Hendra viruses, are zoonotic pathogens that can cause severe respiratory and neurological diseases with high mortality rates in humans. Due to the severity of the disease, the high pandemic potential of these viruses, and the lack of approved treatments, the development of safe and effective medical countermeasures against henipaviruses is a critical priority.

**Methods:**

Here, we evaluate treatment efficacy of defective interfering particles (DIPs)—naturally occurring virus-like particles that lack substantial portions of the viral genome—against henipaviruses in the Syrian hamster model of disease.

**Findings:**

Prophylactic DIP treatment markedly reduced clinical signs and lethality in Syrian hamsters. Single or repeated pre-exposure regimens, starting up to 3 days before challenge, provided protection, while post-exposure treatment was ineffective. DIPs derived from NiV strain Malaysia were most effective against NiV Malaysia but also provided strong protection against the closely related NiV Bangladesh with certain regimens. However, these DIPs offered minimal or no protection against lethality from the more distantly related Hendra virus.

**Interpretation:**

Our data indicate efficacy of DIPs as a pre-exposure prophylactic for henipavirus infection and support a direct mechanism of viral inhibition.

**Funding:**

This work was partially supported by the 10.13039/100000185DARPAINTERfering and Co-Evolving Prevention and Therapy (INTERCEPT) program (DARPA-BAA-16-35), CDC Emerging Infectious Disease Research Core Funds, an appointment to the Research Participation Program at the 10.13039/100000030Centers for Disease Control and Prevention (CDC) administered by the 10.13039/100006229Oak Ridge Institute for Science and Education through an interagency agreement between the 10.13039/100000015U.S. Department of Energy and 10.13039/100000030CDC (K.A.D., S.E.R.), and by 10.13039/100000060NIAID1R01AI151006 (T.E.S).

**Research in context**

**Evidence before this study**

Defective viral genomes, packaged as defective interfering particles (DIPs), are viral particles produced during viral replication that contain defective or truncated genomes, preventing them from replicating independently. First identified over 70 years ago, DIPs were initially recognised in influenza viruses, but they are now known to be present in most RNA viruses. Recent research has focused on harnessing their direct interference properties as a potential treatment modality. DIPs have also been shown to inhibit indirectly through immune system stimulation. In our previous work, we demonstrated production of DIPs derived from the Nipah virus (NiV) genome and showed that concurrent administration of DIPs at time of challenge protects in a stringent animal model of disease.

**Added value of this study**

With DIPs designed based on the Malaysia strain of NiV, we show, using the Syrian hamster model of disease for the Malaysia or Bangladesh strains of NiV, or the closely related Hendra virus (HeV), that increased sequence homology between the DIP source and challenge virus improves protective efficacy. This finding supports direct interference as a key mechanism of DIP-mediated protection for NiV. Additionally, we explore more clinically relevant delivery regimens, focusing on both pre-exposure and early post-exposure treatments.

**Implications of all the available evidence**

Our findings emphasize the importance of engineering DIPs to match the specific virus of interest and suggest that pre-exposure delivery is a promising strategy for protection, especially for a rapidly replicating virus like NiV. While post-exposure treatment may not be as effective for NiV, this could differ for slower-replicating viruses, which may allow for a wider therapeutic window. Understanding these dynamics is crucial for optimising the timing of DIP administration and for integrating them with other prophylactic and post-exposure treatment strategies to improve clinical outcomes.

## Introduction

The henipaviruses (HNV), taxonomically classified within family *Paramyxoviridae*, subfamily *Orthoparamyxoviridae*, comprise a group of emerging high-consequence zoonotic pathogens.^1^^,^^2^ The HNV genome is a single-stranded, negative-sense RNA containing 6 genes from which 9 proteins can be expressed; V, W, and C proteins are encoded within the P open reading frame and translated via mRNA editing and an alternative start codon.^3^ Two HNVs, Nipah virus (NIV) and Hendra virus (HeV) [taxonomically classified within species *Henipavirus nipahens*e and *Henipavirus hendraense*, respectively], are associated with severe respiratory and neurological disease in humans, with case fatality rates ranging 30–80%.^4^ While human HeV infections remain rare, with only 7 recorded cases since its discovery in 1994,^3^ NiV has caused near-annual outbreaks in Bangladesh or India since 2001, and likely circulated unrecognised or misdiagnosed prior to that time, following its initial identification during a large outbreak in Malaysia and Singapore in 1998–1999.^3^^,^^5^ Current treatment options for HNV are limited primarily to supportive care, underscoring the need for effective medical countermeasures such as vaccines and antivirals.^6^^,^^7^

Many viruses are prone to generating defective viral genomes (DVGs) during viral replication.^8^ DVGs are truncated forms of the viral genome that lack the ability to express the full suite of viral proteins and therefore incapable of completing a full replication cycle in the absence of full-length standard virus (STV) that can complement lost functionality.^9^ DVGs, and virus-like particles containing them (termed defective interfering particles [DIPs]), have been ascribed multiple functions in the viral life cycle, like regulating persistence, modulating virulence, and inducing interferon.^8^ However, their most well-characterised defining feature is their ability to directly interfere with STV replication by depleting critical viral and host-cell components. Based on these characteristics, DIPs have been investigated as a promising new class of antivirals for multiple virus species.^10^, ^11^, ^12^, ^13^, ^14^, ^15^, ^16^, ^17^ In previous work, we characterised numerous naturally occurring NiV strain Malaysia (NiV-M) DVGs and developed a production system to incorporated them into DIPs.^18^ We identified several DVGs that reduced NiV-M titres by up to 4 logs in vitro,^18^ and demonstrated evidence of in vivo therapeutic effect in the Syrian hamster model of NiV disease when DVGs are co-administered with virus.^19^

The overall aim of this study was to determine prophylactic and postexposure in vivo efficacy of intranasally delivered DIPs in treating the highly pathogenic HNVs, NiV and HeV. We first assessed the efficacy of DIP treatment against NiV-M when given up to 7 days before or 1 day after challenge in the Syrian hamster model. We then characterised clinical progression and lethality in Syrian hamsters following infection with NiV strain Bangladesh (NiV-B) or HeV and evaluated DIP effectiveness using the same dosing schedules in these heterologous challenge models. We found that pre-exposure DIP treatment was highly effective at reducing both appearance of clinical signs and lethality in Syrian hamster HNV disease models, although greater genetic distance between the challenge virus and the parental origin of the DVGs was associated with poorer clinical outcomes. Together, these data add to the growing evidence supporting the use of DVGs as a treatment approach to control HNVs and other viral infections.

## Methods

### Biosafety

All work with infectious virus or infected animals was conducted in a biosafety level 4 (BSL-4) laboratory at the Centers for Disease Control and Prevention (CDC) following established BSL-4 standard operating procedures approved by the Institutional Biosafety Committee. All recombinant virus work was approved by the CDC Institutional Biosafety Committee.

### Cells and viruses

Vero-E6 (ATCC, CRL-1586, RRID:CVCL_057), Vero (ATCC, CCL-81, RRID:CVCL_0059), and BSR-T7/5 (RRID:CVCL_RW96) cells were cultured in Dulbecco’s modified Eagle’s medium (DMEM) supplemented with 5% (v/v) foetal calf serum, non-essential amino acids, 1 mM sodium pyruvate, 2 mM L-glutamine, 100 U/mL penicillin, and 100 μg/mL streptomycin (all from Gibco). NiV strain Malaysia (NiV-M; isolate 199901924; GenBank accession no. AF212302; CDC reference #813744) was obtained from a 1999 clinical specimen (human cerebrum), passaged once on Vero-E6 cells for isolation and further amplified once on Vero cells. NiV strain Bangladesh (NiV-B; isolate 200401066; GenBank accession no. AY988601; CDC reference #813747) was obtained from a 2004 clinical specimen (human throat swab) from a lethal case, passaged once on Vero-E6 cells for isolation and further amplified once on Vero cells. HeV strain 9409-30-1800 (GenBank accession no. MN062017; CDC reference #814127) was obtained from a 1994 clinical specimen (horse lung), passaged 3 times on Vero-E6 cells for isolation and further amplified once on Vero cells. Viral titres were calculated as 50% tissue culture infective dose (TCID_50_) in Vero cells.^20^ All viral stocks were verified by NGS and confirmed to be mycoplasma-free.

### DIP production

DIPs were produced as previously described.^18^ Briefly, DIPs were generated using a modified version of the established NiV reverse genetics rescue system,^21^ in which a plasmid transcribing the DVG sequence, along with support plasmids expressing N, P, and L proteins, was transfected into BSR-T7/5 cells. After 48 h, the transfected BSR-T7/5 monolayer was overlayed with Vero cells and infected at a multiplicity of infection (MOI) of 0.01 (based on Vero cell number only) with a recombinant NiV-M expressing ZsGreen1 fluorescent protein (rNiV-M/ZsG) in order to supply the additional viral proteins required for DIP assembly and release.^22^ At 7 days post transfection (5 days post viral infection [dpi]), the supernatant was removed and clarified by low-speed centrifugation (500×*g*, 10 min). To deplete infectious rNiV-M/ZsG from the DIP product, samples were treated with 100 mJ/cm^2^ of ultraviolet radiation (CX-2000 Crosslinker; UVP) in a 6-well plate (1 mL sample/well) immediately prior to use. This dose has previously been shown to result in breakage of the longer full-length rNiV-M/ZsG RNA genomes but leave intact the shorter DVGs incorporated into the DIP.^18^ Removal of infectious rNiV-M/ZsG was verified by infecting cells with the active DIP product and examining cells for absence of ZsG expression 7 dpi. DIPs were quantified using DVG-specific digital droplet RT-qPCR assays. On average, this production system resulted in a final concentration of approximately 10^9^ DI genome copies/mL.

### Ethical statement and hamster studies

All animal experiments were approved by the CDC Institutional Animal Care and Use Committee (IACUC; #2956, 3220) and the United States Army Medical Research and Development Command (USAMRDC) Animal Care and Use Review Office (ACURO) and performed in an AAALAC-International approved facility. Data are based on 6 independent hamster studies, all using HsdHan:AURA Syrian hamsters (6 weeks old; Envigo no. 8903F or 8903M). Group sizes were 8–10 for each treatment/control cohort, with an equal proportion of male and female animals. As studies were exploratory in nature, and effect size and standard deviation were not known for power analysis, groups sizes were determined based on experience with the disease model and confirmed to be within acceptable range of degrees of freedom using the ‘resource equation’ approach. Investigators were not blinded to cohort allocation during data acquisition. Inclusion criteria included healthy age- and sex-matched hamsters, with no prior experimental manipulation, no clinical signs of disease at baseline, and sourced from an approved vendor; no animals were excluded. Clinical data comprised objective measures (e.g., weight, body temperature) and standardised scoring systems specific to these disease models, applied uniformly across all groups by experienced personnel.

DIP were administered intranasally (IN; 100 μL total volume divided between the nares) using a pipette. Mock treated hamsters (no DIP controls) were inoculated with DMEM IN. Hamsters were challenged with virus either IN (100 μL total volume divided between the nares), or intraperitoneally (IP; 500 μL total volume split bilaterally). Microchip transponders (BMDS IPTT-300) were placed subcutaneously in the interscapular region at ∼4 days prior to study start date to allow for individual identification and for body temperature assessment. Baseline weights were taken −1 dpi, and hamsters were assessed daily post inoculation to record weight change, body temperature and clinical score. Animals were scored by the following criteria: quiet dull responsive, hunched back/ruffled coat, hypoactivity, mild neurological signs—each 2 points; abnormal breathing (e.g., increased respiratory rate, dyspnoea), hypothermia (<34 °C), moderate neurological signs—each 5 points; paralysis, frank haemorrhage, moribund, weight loss >25% of baseline (measured at −1 dpi), severe neurological signs—each 10 points. Neurological signs were classified as: mild–abnormal gait or movement, mild (∼0–30° from vertical) or sporadic head tilt; moderate–tremors, ataxia, circling, absence seizures, moderate (∼>30–90° from vertical) and persistent head tilt while retaining ability to ambulate, eat, and drink; severe—limb paralysis, tonic clonal seizure, inability to right, severe (>90° from vertical) head tilt. Hamsters were euthanised with isoflurane vapour when they met euthanasia criteria (score ≥10) or at the completion of the study (28 dpi).

### In vitro inhibition studies

DIP inhibition assays were performed as previously described.^18^ Briefly, an active and inactive stock of each tested DIP was generated. Active DIPs were made by treating the initial clarified production supernatant (containing infectious rNIV-M/ZsG) with 100 mJ/cm^2^ of ultraviolet radiation, depleting the infectious rNIV-M/ZsG component but leaving the DVG intact. Inactive DIPs were treated with 10 × 240 mJ/cm^2^ of UV radiation to deplete both rNIV-M/ZsG genome and DVGs. For each DIP tested, 2 × 10^3^ TCID_50_/mL of virus (NiV-M, NiV-B, or HeV) was mixed with 1 mL of active or inactive DIP stock, and then 500 μL of the mixture was used to treat 5 × 10^4^ Vero cells/well of a 24-well plate (final MOI of 0.01). At 2 dpi, supernatant was collected and viral titres were determined as TCID_50_ in Vero cells.^20^ Fold reduction in viral titres for each DIP was calculated by dividing titres from cells treated with inactive DIPs by titres from cells treated with active DIPs.

### Reverse-transcription quantitative polymerase chain reaction

DIP concentrations were quantified using a digital droplet reverse-transcription quantitative polymerase chain reaction (ddRT-qPCR) assay. A 20 × mix of DVG-specific primers and probes (18 μM each primer and 5 μM probe) were used in conjunction with 1-Step RT-ddPCR Advanced Kit for Probes and Droplet Generation Oil for Probes (both from Bio-Rad). Duplex quantitative reverse-transcription polymerase chain reaction (RT-qPCR) reactions were set up and run as per manufacturers’ conditions, with droplets generated using QX200 Droplet Generator (Bio-Rad). Results were analysed using QX200 Droplet Digital PCR System, and quantification data were generated using QuantaSoft Software (both from Bio-Rad). NiV-M tissue and oral swab viral RNA (vRNA) loads were quantified using a RT-qPCR assay targeting the N gene sequence (NiV Fwd—CTG GTC TCT GCA GTT ATC ACC ATC GA; NiV Rev—ACG TAC TTA GCC CAT CTT CTA GTT TCA; NiV Probe—FAM-CAG CTC CCG ACA CTG CCG AGG AT-BHQ1; all 5ʹ to 3ʹ; all from IDT), with copy numbers normalised to 18S RNA values using a commercial endogenous control assay (Thermo Fisher). Genome copy numbers were determined using standards prepared from in vitro-transcribed N RNA. Viral RNA was extracted from homogenised tissue samples and whole blood using the MagMAX-96 Total RNA Isolation Kit (Thermo Fisher) on a 96-well ABI MagMAX extraction platform with a DNase-I treatment step according to manufacturer’s instructions.

### Statistics and graphing

All graphs and statistical analyses were created in GraphPad Prism (v10). Significance for differences in body weight and temperature were calculated using unpaired t test, multiple comparisons using false discover rate method of Benjamini, Krieger and Yekutieli. Significance in Kaplan–Meier curves was calculated using the log-rank (Mantel–Cox) test. Significances for differences between DIP variants in reducing viral titres in vitro and for differences in vRNA loads in select tissues (as calculated by RT-qPCR) were calculated using unpaired t test, multiple comparisons using false discover rate method of Benjamini, Krieger and Yekutieli.

### Role of funders

Funders had no input on study design, data collection, data analyses, data interpretation or writing of report.

## Results

### Detection of DIPs following intranasal delivery is limited to lung tissue

**Fig. 1:**
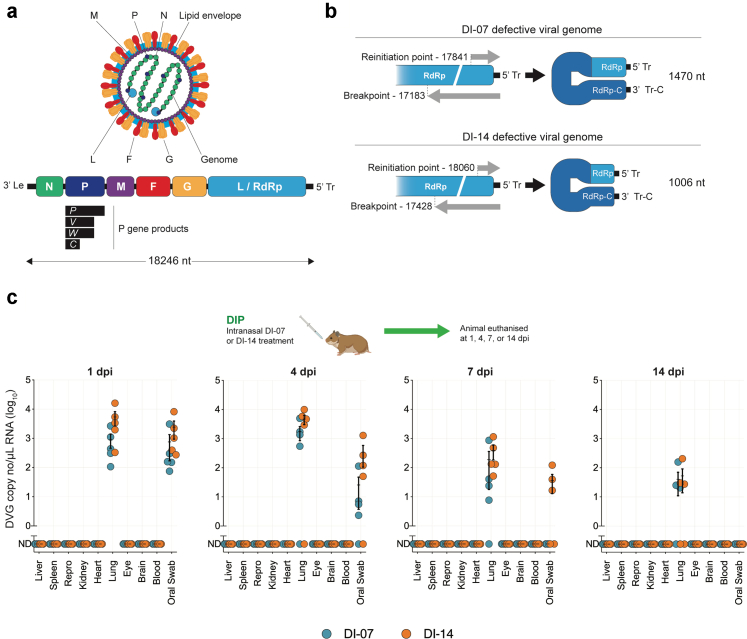
**Nipah virus and defective viral genome genomic architecture, and persistence of defective viral genomes in vivo. (a)** Virion and viral genome schematic of Nipah virus (NiV). Viral genome is represented in the genomic polarity: 3′Le, leader sequence; N, nucleoprotein; P, phosphoprotein; M, matrix protein; F, fusion protein; G, glycoprotein; L, viral RNA-dependent-RNA-polymerase (RdRp); 5′Tr, trailer sequence. **(b)** Genome schematics of the copyback defective viral genomes (DVG) for DI-07 and DI-14 incorporated into defective interfering particles (DIPs). Breakpoint and reinitiation point nucleotide numbers are in the context of genomic polarity (based on NiV strain Malaysia; GenBank accession no. AF212302). RdRp-C and Tr-C are the complementary nucleotide sequences of the RdRp and trailer sequences, respectively. Within the copyback DVG, the complementary region lengths for DI-07 and DI-14 are 406 nt and 187 nt, respectively. **(c)** Persistence of DI-07 (teal) and DI-14 (orange) RNA in Syrian hamsters after intranasal inoculation with 10^8^ DIPs (n = 5 animals per DIP treatment per timepoint) determined by RT-qPCR. Repro indicates testis or ovary. Each circle represents an individual animal; error bars indicate the mean and standard deviation. dpi, days post inoculation.

DVGs require STV to replicate, so their persistence in vivo is limited before they are degraded by standard cellular pathways that recognise and remove aberrant molecules. Previous studies of other DVGs have suggested that, in the absence of STV, DVGs could persist 1–3 weeks.^23^^,^^24^ To determine optimal prophylactic treatment timing for NiV DIP administration, we first assessed persistence of DVGs following delivery. Syrian hamsters (total number used = 40) were inoculated IN with 10^8^ DIPs containing either DI-07 (n = 20) or DI-14 (n = 20). DI-07 (1470 nt) and DI-14 (1006 nt) are trailer copyback variants based on the NiV-Malaysia genome (Fig. 1a), with unique break and reinitiation points (Fig. 1b). These DVGs were previously identified as the most promising treatment candidates based on their ability to inhibit NiV-M in vitro and in vivo.^18^^,^^19^

Five hamsters from each cohort were serially euthanised 1, 4, 7, and 14 days post infection (dpi). Aligning with previous data supporting the safety of DIP treatment, no adverse signs were noted in any animals throughout the 14 day study period.^19^ For both groups, DVG RNA was not detected in any tissue except lung, reflecting IN delivery to respiratory tract (Fig. 1c). DVG RNA levels remained consistent in lung from 1 to 4 dpi, decreased 1–2 logs at 7 dpi, and continued to drop 1–1.5 logs by 14 dpi. DI-14 RNA levels were consistently higher than DI-07 RNA across the 14 days study period. In oropharyngeal swabs, DVG RNA levels were highest at early timepoints (1 and 4 dpi), declining 7 dpi and absent by 14 dpi. As detection of DVG RNA in oropharyngeal swabs cannot be due to DIP shedding, these results likely represent residual DIP remaining at the mucosal surface or from DVG-containing cells mechanically removed from the mucosa by the swabbing action. Similarly to lung tissue, DI-07 RNA levels in oropharyngeal swabs were undetectable 7 dpi, whereas 3 of 5 (60%) of the animals given DI-14 had detectable DVG RNA in oropharyngeal swabs 7 dpi (Fig. 1c). Overall, based on higher abundance and more prolonged detection of DI-14 DVG RNA, we chose to concentrate on DIPs containing DI-14 in subsequent prophylactic efficacy studies.

### Prophylactic DIP treatment based on NiV-M-derived DVGs protects against lethal disease after homologous challenge in Syrian hamsters

**Fig. 2:**
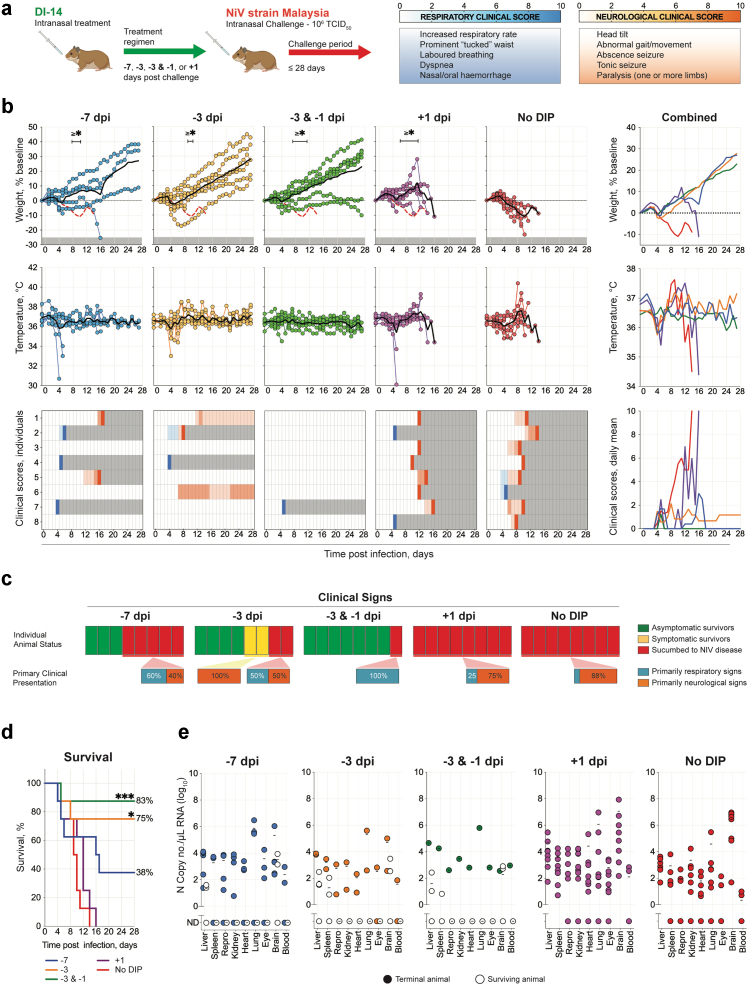
**Intranasal challenge with NiV strain Malaysia after prophylactic DIP treatment. (a)** Syrian hamsters (n = 8 per group) were treated intranasally (IN) with 10^8^ DIPs containing DVG DI-14: once 7 (−7 dpi) or 3 days (−3 dpi) prior to challenge; twice 3 and 1 days (−3 & −1 dpi) prior to challenge; or once 1 day (+1 dpi) post challenge; or mock-treated with DMEM (no DIP). At day 0, hamsters were challenged IN with 10^6^ TCID_50_ of NiV strain Malaysia (NiV-M) and followed for 28 days. **(b)** Graphs showing the percent weight change from baseline (taken at −1 dpi) and body temperature, with each circle representing an individual animal and the solid black line indicating the mean. Clinical signs (scored daily from 0 to 10, with typical clinical signs and scale bar noted in the top right of figure). Severity is indicated by increasing intensity of blue (respiratory signs) or red (neurological signs). Animals scoring ≥10 were humanely euthanised; any animals that succumbed to disease prior to euthanasia were allocated a score of 10. Grey boxes indicate the end of monitoring/scoring due to euthanasia/death. Also shown are graphs representing daily means of weight, body temperature, and clinical scores, combined. **(c)** Breakdown of asymptomatic (green), symptomatic (displayed clinical signs for at least 1 day during study; yellow), and terminal (red) animals per treatment group. For symptomatic and terminal animals, a breakdown of their primary clinical disease presentation is included: respiratory (blue) or neurological (orange). **(d)** Survival of hamsters in each group. Significance calculated by log-rank (Mantel–Cox test): ✱✱✱, *p* ≤ *0.001*; ✱, *p* ≤ *0.05*. **(e)** Tissue NiV-M vRNA loads determined by RT-qPCR. Repro indicates testis or ovary. Each circle represents an individual animal, and the solid black line indicates the mean. Solid circles indicate animals that succumbed to infection and open circles indicate survivors.

Previous work demonstrated that DIP treatment simultaneous with challenge greatly improved clinical outcome in the NiV Syrian hamster disease model.^19^ To further investigate the clinical utility of DIPs, we next assessed the efficacy of NiV-M-derived DIPs following pre- and post-challenge delivery. As DVG RNA notably declines 7 dpi (Fig. 1c), we chose 7 days as the maximum pre-challenge treatment period. Furthermore, as onset of NiV-M disease and time-to-death can be rapid in hamsters,^19^ we chose 1 day as the maximum post-challenge treatment period. Syrian hamsters (total number used = 40, n = 8 per treatment group) were inoculated IN with 10^8^ DIPs containing DI-14, either once 7 days before viral challenge (−7 dpi); once 3 days before challenge (−3 dpi); twice, 3 days and 1 day before challenge (−3 & −1 dpi); or once 1 day post challenge (+1 dpi). Mock-treated hamsters (no DIP) were treated IN with vehicle only (DMEM), with one dose 7 days before challenge. All hamsters were challenged IN with 10^6^ TCID_50_ of NiV-M on day 0, and followed until 28 dpi (Fig. 2a).

Mock-treated (no DIP) control animals exhibited weight loss beginning 2–3 dpi and elevated body temperatures at end point (associated with severe neurological signs, predominantly persistent seizures) from 8 dpi onwards (Fig. 2b). Clinical signs of NiV disease were observed in all mock-treated animals ≥1 day prior to their meeting euthanasia criteria or succumbing to disease. One animal (12.5%) displayed primarily respiratory signs and the remaining 7 animals (87.5%) developed severe neurological signs (Fig. 2c); infection was 100% lethal in all animals by 14 dpi (mean time to death [MTTD] = 9.5 days) (Fig. 2d). In contrast, all DIP-treated animals maintained higher mean body weights than mock-treated animals during the acute disease phase (∼6–11 dpi) and had lower incidence of clinical signs and improved survival rates.

DIP treatment was most efficacious when administered twice (−3 & −1 dpi); 7 of 8 (88%) hamsters in this treatment group survived viral challenge and remained asymptomatic until study end. A single DIP treatment 3 days prior to challenge (−3 dpi) also provided considerable protection, with only 2 of 8 (25%) animals succumbing to infection (MTTD = 6.5 days). Of the 6 survivors, 4 (50%) remained asymptomatic until study end. The remaining 2 animals developed neurological signs, one displaying a persistent mild head tilt (∼30° left) from 12 dpi until study end, and one displaying a persistent moderate head tilt (∼45° left) accompanied by mild ataxia and hind limb paresis from 7 dpi until study end (Fig. 2b). More limited protection was seen in animals treated 7 days prior to challenge (−7 dpi); 3 animals (37.5%) survived and remained asymptomatic, but all 5 remaining animals exhibited either severe respiratory (37.5%) or neurological (25%) disease and reached end point criteria (MTTD = 9.2 days). Post-exposure treatment (+1 dpi) offered no protection from lethality, with all animals succumbing by 16 dpi (MTTD = 10.8 days). In all animals that succumbed to disease (either no DIP control or DIP-treated hamsters), viral RNA (vRNA) was widely detected in all tissues, with highest levels typically observed in lung and brain (Fig. 2e). In contrast, vRNA was generally undetectable in tissues from DIP-treated animals that survived challenge. In a subset of DIP-treated survivors, vRNA was detected but typically at reduced levels (∼2–4 logs) and limited to liver, spleen, and brain (Fig. 2e).

### In vitro DIP-mediated inhibition against HNV strains and species decreases with increasing heterogeneity from parental virus

**Fig. 3:**
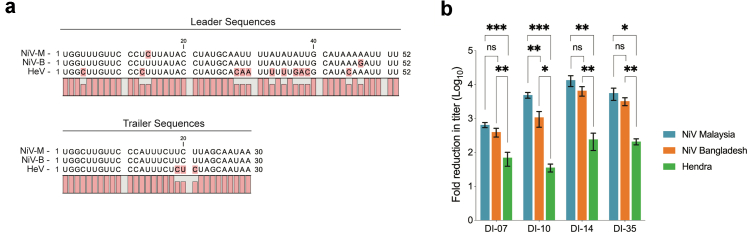
**Henipavirus leader and trailer sequence homology and in vitro efficacy of DIPs against other henipaviruses. (a)** Alignments of the genomic sense leader and trailer sequence of NiV-M (GenBank accession no. AF212302), NiV strain Bangladesh (NiV-B) (GenBank accession no. AY988601), and Hendra virus (HeV) (GenBank accession no. MN062017). Nucleotide differences from consensus are highlighted in red. **(b)** In Vero cells treated with DIPs containing the DVGs DI-07, DI-10, DI-14, or DI35, represented are fold reductions in titres for NiV-M (blue), NiV-B (orange), or HeV (green) compared to untreated cells. Bars represent the mean, and error bars represent the standard deviation. Significant differences in levels of inhibition between the DIP + virus groups were calculated by multiple t-test. ✱✱✱, *p* ≤ *0.001*; ✱✱, *p* ≤ *0.01*; ✱, *p* ≤ *0.05*; ns, not significant.

Given the promising prophylactic results observed with the NiV-M-derived DIPs against homologous challenge virus (NiV-M), we sought to explore the specificity of this inhibition to assess the broader applicability of this approach. The three pathogenic HNVs known to cause severe disease in humans are NiV-M, NiV-B, and HeV. Whilst they all share high sequence heterogeneity, minor differences are noted in the promoter regions, specifically the leader and trailer sequences at the genome termini, which are critical for regulating transcription and replication processes (Fig. 3a). Given that efficient replication of DVGs is important in mediating their inhibitory effect, we sought to determine the ability of NiV-M-derived DIPs to inhibit both NiV-B and HeV.

We performed in vitro assessment using DIPs containing one of four NiV-M genome sequence-derived DVGs: DI-07, DI-14, or DI-35 (all copyback variants); or DI-10 (deletion variant). Previously described DVGs DI-10 and DI-35^18^ were included because both have previously demonstrated protective efficacy against NiV-M infection in the hamster model.^19^ DIPs were mixed with wild-type NiV-M, NiV-B, or HeV at a ratio of 5000:1 (DIP:virus) and added to cell monolayers, and viral titres were determined 48 h later. For NiV-M, the greatest fold reduction in titre compared to untreated controls was observed using DI-14 (mean reduction 4.13 logs), but all DIP variants reduced viral titres (Fig. 3b). Consistent with their ability to reduce NiV-M titres, all DIP variants equivalently reduced NiV-B titres by 2.5–3.5 logs, except for DI-10 which inhibited NiV-B significantly (*p* = 0.004, calculated with multiple t-test) less than NiV-M. The 2 nt differences in the leader between NiV-M and NiV-B may account for the decreased inhibition by DI-10, since it is a deletion DIP, and both trailer and leader promoters are utilised during DVG replication. Moreover, all tested DIPs reduced HeV titres by 1.5–2.4 logs, but inhibition efficacies were significantly (all *p* > 0.05, calculated with multiple t-test) reduced compared to the DIPs’ ability to inhibit NiV-M and NiV-B, likely due to impaired recognition of the DIP promoter by the HeV replication complex. Overall, DVG DI-14 demonstrated the strongest inhibition against all viruses, with mean fold titre reductions of 4.13, 3.82, and 2.38 logs of NiV-M, NiV-B, and HeV titres, respectively.

### Syrian hamster disease model for DIP assessment against NiV-B and HeV

Because NiV-M-derived DIPs exhibited in vitro differences in inhibiting HNV strains and species, we next wanted to determine whether these observations translated in vivo. We previously characterised a NiV-M Syrian hamster disease model, utilising a detailed clinical scoring system incorporating multiple clinical variables to document respiratory and neurological disease progression^19^ for use in DIP pre-clinical assessment studies. To evaluate DIP efficacy against other HNVs, here we characterised analogous Syrian hamster disease models of NiV-B and HeV.

**Fig. 4:**
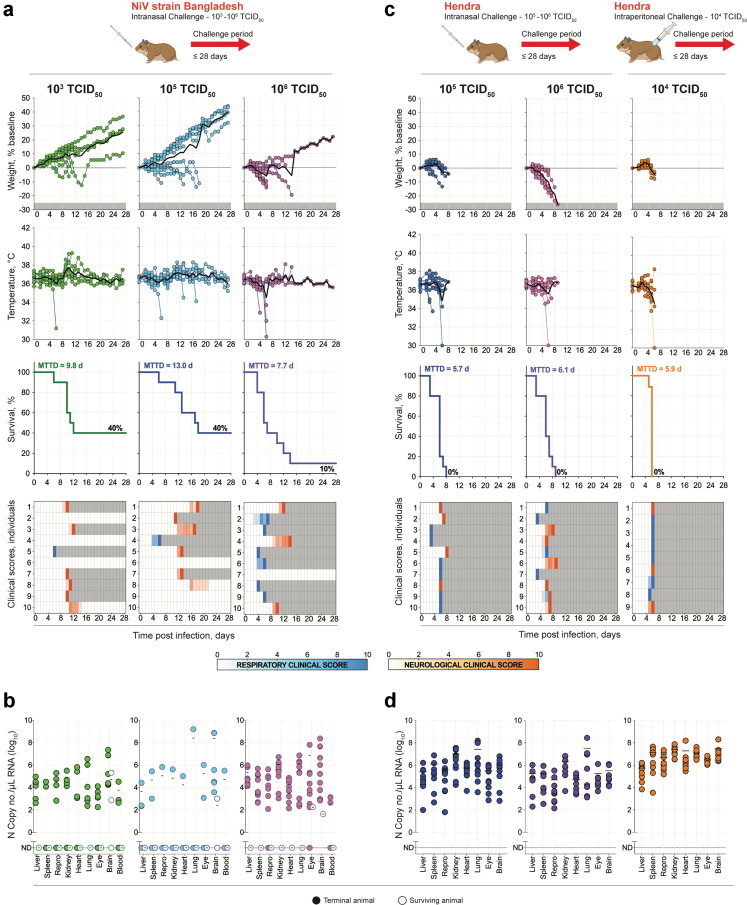
**Characterisation of disease caused by NiV strain Bangladesh or HeV in the Syrian hamster model using different doses and route of inoculation. (a)** Syrian hamsters (n = 10 per group) were challenged intranasally (IN) with either 10^3^ (green), 10^5^ (blue), or 10^6^ (purple) TCID_50_ of NiV strain Bangladesh (NiV-B), and followed for 28 days. Graphs show the percent weight change from baseline (taken −1 dpi), changes in body temperature, survival, and clinical signs (scored from 0 to 10). Severity is indicated by increasing intensity of blue (respiratory signs) or red (neurological signs). Animals scoring ≥10 were humanely euthanised; any animals that succumbed to disease prior to euthanasia were allocated a score of 10. Grey boxes indicate the end of monitoring/scoring due to euthanasia/death. **(b)** Tissue NiV-B vRNA loads determined by RT-qPCR. Each circle represents an individual animal, and the solid black line indicates the mean. Animals that succumbed to infection are shown as solid circles; surviving animals are shown as open circles. **(c)** Syrian hamsters (n = 10 per group) were challenged intranasally (IN) with either 10^5^ (dark blue) or 10^6^ (pink) TCID_50_, or challenged intraperitoneally (IP) with 10^4^ (orange) TCID_50_ of HeV and followed for 28 days. Graphs show percent weight change from baseline (taken −1 dpi), changes in body temperature, survival, and clinical signs (scored from 0 to 10). Severity is indicated by increasing intensity of blue (respiratory signs) or red (neurological signs). Animals scoring ≥10 were humanely euthanised; any animals that succumbed to disease prior to euthanasia were allocated a score of 10. Grey boxes indicate the end of monitoring/scoring due to euthanasia/death. **(d)** Tissue HeV vRNA loads determined by RT-qPCR. Individual animals are represented as circles, and the solid black line represents the mean. Solid circles represent animals that succumbed to infection, and open circles represent survivors. For vRNA, repro indicates testis or ovary. For survival curves, dashed grey line represents the survival curve for NiV-M and the equivalent dose and route. MTTD, mean time to death.

We assessed IN inoculation in hamsters with NiV-B challenge doses of 10^3^, 10^5^, and 10^6^ TCID_50_ (n = 10 per dose, as with NiV-M^19^). Like with NiV-M,^19^ higher NiV-B doses resulted in more symptomatic animals and higher lethality, with a clinical course characterised by greater weight loss and more numerous hypothermic and hyperthermic events than when using lower doses (Fig. 4a). The highest dose of NiV-B (10^6^ TCID_50_) was lethal in 9 of 10 (90%) hamsters, whereas the lower doses were both lethal in 6 of 10 (60%). With 10^6^ TCID_50_, severe respiratory disease was seen in 6 animals (60%) occurring 3–6 dpi; severe neurological disease (e.g., circling, hyperactivity, ataxia, and/or seizures) was seen in 3 animals (30%) 9–14 dpi. With the two lower challenge doses (10^3^ and 10^5^ TCID_50_), severe respiratory disease was apparent 4–6 dpi and only observed in 10% of animals, whereas the most prominent clinical signs were neurological (50% for both doses) and appeared later in the course of infection (9–18 dpi). Tissue vRNA dissemination patterns and loads were similar in all animals that succumbed to NiV-B challenge regardless of dose (Fig. 4b). Generally, vRNA was undetectable in surviving animals (28 dpi), although persistence in the brain and eye was noted in some.

We then evaluated comparable HeV challenge route and doses as used in our previous studies in hamsters (10^6^ TCID_50_ IN and 10^4^ TCID_50_ IP challenge; n = 9–10 per dose).^19^^,^^25^ As reports in hamsters indicate higher lethality after HeV than NiV IN challenge,^26^ we also used a lower dose (10^5^ TCID_50_ IN) to increase chances of a more tractable model displaying a broader range of symptoms and a wider MTTD. With all routes and doses, infection was uniformly lethal within approximately 6 dpi independently of dose and route (Fig. 4c). Weight loss associated with the onset of clinical signs was observed starting 2–4 dpi, and the appearance of severe clinical signs was rapid, with animals often progressing from asymptomatic to reaching euthanasia criteria within 24 h. As with NiV disease, the earliest observed clinical signs were respiratory, although, in contrast to NiV, neurological signs appeared much earlier in the disease course (∼5 dpi compared to ∼8 dpi for NiV). Independent of dose and route, the distribution of animals succumbing with severe respiratory versus neurological signs was approximately 1:1. HeV vRNA was detected in all tissues assessed, with levels typically higher in IP-inoculated than IN-inoculated hamsters despite a 10- or 100-fold lower virus dose; mean viral loads by tissue type were on average 17.3 fold-higher (range, 0.3–70.9) (Fig. 4d). In IN-inoculated animals, vRNA levels were highest in the lungs, whereas IP-challenged animals had highest amounts in the brain. Comparing NiV and HeV, proportional vRNA loads between tissue types were similar, although total vRNA quantities in HeV-infected animals tended to be higher than in NiV-infected hamsters for individual tissue types.

### DIP-mediated protection against HNV disease in the Syrian hamster model decreases against strains or species with greater genetic distance from the parental strain

**Fig. 5:**
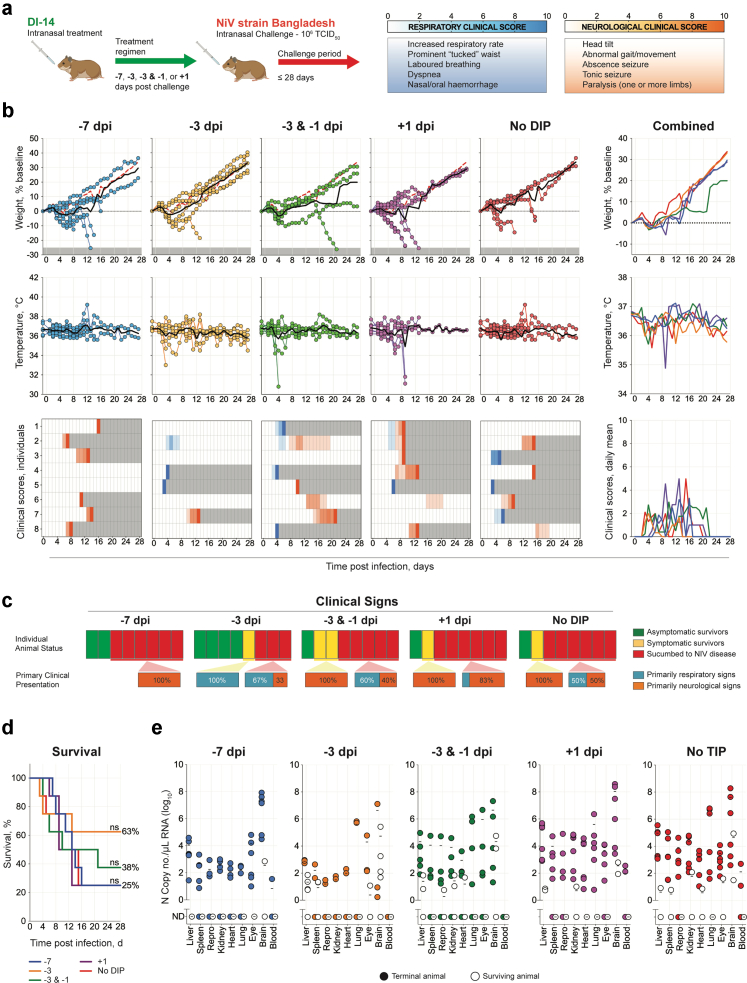
**Intranasal challenge with NiV strain Bangladesh after prophylactic DIP treatment. (a)** Syrian hamsters (n = 8 per group) were treated intranasally (IN) with 10^8^ DIPs containing DI-14: once 7 (−7 dpi) or 3 days (−3 dpi) prior to challenge; twice 3 and 1 days (−3 & −1 dpi) prior to challenge; or once 1 day (+1 dpi) post challenge; or mock-treated with DMEM (no DIP). At day 0, hamsters were challenged IN with 10^6^ TCID_50_ of NiV-B and followed for 28 days. **(b)** Graphs showing percent weight change from baseline (taken −1 dpi) and body temperature, with individual animals represented as circles and solid black line representing the mean. Clinical signs (scored daily from 0 to 10, with typical clinical signs and scale bar noted in the top right of figure). Severity is indicated by increasing intensity of blue (respiratory signs) or red (neurological signs). Animals scoring ≥10 were humanely euthanised; any animals that succumbed to disease prior to euthanasia were allocated a score of 10. Grey boxes indicate the end of monitoring/scoring due to euthanasia/death. Also shown are graphs representing daily means of weight, body temperature, and clinical scores, combined. **(c)** Breakdown of asymptomatic (green), symptomatic (displayed clinical signs for at least 1 day during study; yellow), and terminal (red) animals per treatment group. For symptomatic and terminal animals, a breakdown of their primary clinical disease presentation is included: respiratory (blue) or neurological (orange). **(d)** Survival of hamsters in each group. Significance calculated by log-rank (Mantel–Cox test): ns, not significant. **(e)** Tissue NiV-B vRNA loads determined by RT-qPCR. Repro indicates testis or ovary. Each circle represents an individual animal, and the solid black line indicates the mean. Solid circles represent animals that succumbed to infection, and open circles represent survivors.

Using the hamster models for NiV-B and HeV described above, we then tested the efficacy of NiV-M-derived DIPs against heterologous challenge viruses. To evaluate DIP efficacy against NiV-B disease, Syrian hamsters (n = 8 per treatment group) were treated IN with 10^8^ DIPs containing DI-14 once −7 dpi; once −3 dpi; twice, at −3 & −1 dpi; or once +1 dpi. Mock-treated hamsters (n = 7) were given DMEM once −7 dpi (no DIP). All animals were then challenged IN with 10^6^ TCID_50_ of NiV-B on day 0, and followed until 28 dpi (Fig. 5a). In DIP-treated animals, independently of treatment timing, no notable differences in mean body weight or temperature were observed compared to mock-treated animals (Fig. 5b). However, animals treated once at −3 dpi were more likely to survive (62.5%) and remain asymptomatic throughout the study (50%) than the no-DIP controls (Fig. 5c and d). All other treatments resulted in outcomes similar to the no-DIP controls in terms of numbers of symptomatic animals and overall survival. As with the NiV-M-challenged animals, vRNA tissue levels in animals that succumbed to NiV-B infection were comparable between DIP-treated and no-DIP animals, independently of treatment timing (Fig. 5e). vRNA was detected in a subset of survivors at study end (28 dpi; both treated and untreated), but at lower levels than in animals that succumbed, suggesting inhibition of virus replication by the host response or DIPs.

**Fig. 6:**
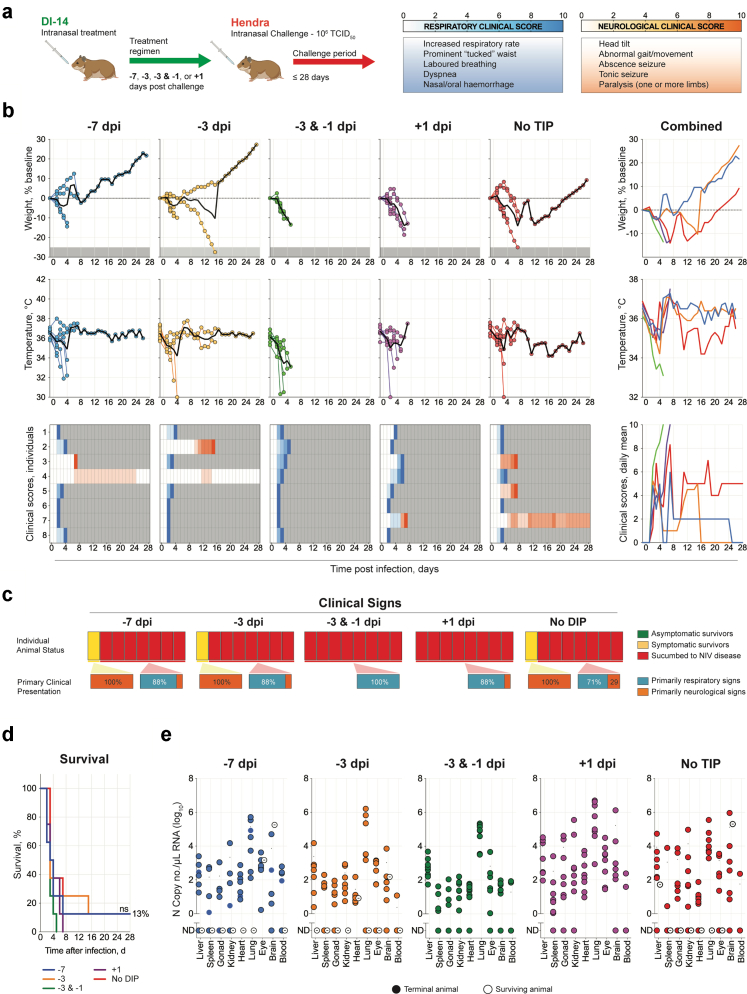
**Intranasal challenge with Hendra virus after prophylactic DIP treatment. (a)** Syrian hamsters (n = 8 per group) were treated IN with 10^8^ DIPs containing DVG DI-14 once 7 (−7 dpi) or 3 days (−3 dpi) prior to challenge; twice 3 and 1 days (−3 & −1 dpi) prior to challenge; or once 1 day (+1 dpi) post challenge; or mock-treated with DMEM (no DIP). At day 0, hamsters were challenged IN with 10^6^ TCID_50_ of HeV and followed for 28 days. **(b)** Graphs showing the percent weight change from baseline (taken −1 dpi) and body temperature. Each circle represents an individual animal, and the solid black line indicates the mean. Clinical signs (scored daily from 0 to 10, with typical clinical signs and scale bar noted in the top right of figure). Severity is indicated by increasing intensity of blue (respiratory signs) or red (neurological signs). Animals scoring ≥10 were humanely euthanised; any animals that succumbed to disease prior to euthanasia were allocated a score of 10. Grey boxes indicate the end of monitoring/scoring due to euthanasia/death. Also shown are graphs representing daily means of weight, body temperature, and clinical scores, combined. **(c)** Proportions of asymptomatic (green), symptomatic (yellow; clinical signs for ≥1 day), and terminal (red) animals per treatment group. For symptomatic and terminal animals, the primary clinical presentation is categorised as respiratory (blue) or neurological (orange). **(d)** Survival of hamsters in each group. Significance calculated by log-rank (Mantel–Cox test): ns, not significant. **(e)** Tissue HeV vRNA loads determined by RT-qPCR. Gonad indicates testis or ovary. Each circle represents an individual animal, and the solid black line indicates the mean. Solid circles represent animals that succumbed to infection, and open circles represent survivors.

We repeated the DIP treatment study with HeV; all hamsters (n = 10 per group) were challenged IN with 10^6^ TCID_50_ of virus on day 0 and followed until 28 dpi (Fig. 6a). Here, independently of treatment status or length, animals generally displayed rapid-onset weight loss associated with appearance of clinical signs around 2–4 dpi (Fig. 6b). No animal remained asymptomatic for the study period, with the majority succumbing to rapid-onset respiratory disease 2–6 dpi (Fig. 6c). Neurological signs were observed in animals that survived beyond the acute respiratory disease window, with most succumbing by 14 dpi. The 3 surviving animals all displayed neurological clinical signs at some point throughout the study period. In animals that succumbed, MTTD was similar independently of treatment status: −7 dpi, 3.4 days; −3 dpi, 4.3 days; −3 & −1 dpi, 3.0 days; +1 dpi, 4.1 days; and no DIP, 4.3 dpi (Fig. 6d). As with both strains of NiV, tissue HeV vRNA levels were comparable in all animals that succumbed, with all surviving animals also having relatively high vRNA levels in the brain (Fig. 6e).

## Discussion

DIPs can protect against lethal outcome and disease in animal models when co-administered with challenge virus, as shown for NiV, influenza A virus (IAV), severe acute respiratory syndrome coronavirus 2 (SARS-CoV-2), and Zika virus (ZIKV).^10^^,^^11^^,^^14^^,^^19^^,^^27^^,^^28^ Here, we advance the development of DIPs for the treatment of NiV infection by investigating clinically relevant pre- and post-exposure delivery regimens. Notably, our data demonstrate that pre-exposure DIP treatment can effectively inhibit NiV, with protection mediated through a direct interference mechanism. Like our earlier studies of simultaneous delivery, pre-exposure DIP treatment starting as early as 3 days prior to challenge with homologous virus improved clinical outcomes. Similar data have been reported for IAV when comparing simultaneous and pre-exposure (3 days prior) DIP treatments.^14^^,^^15^^,^^29^^,^^30^ The extent of prophylactic protection tends to closely reflect the kinetics of in vivo DIP persistence. In hamsters, we showed that DVG quantities remained high in lung tissue up to 4 days post treatment but began to wane on day 7. These kinetics closely correlate with observed DIP efficacy, where treatment 3 days prior to challenge resulted in a better outcome than treatment 7 days prior to challenge. Contrasting this, DVG RNA from an IAV DIP remained detectable for up to 3 weeks post treatment in mice; in this same model, treatment 7 days prior to challenge remained uniformly protective against IAV lethality and disease.^23^ Other variables such as disease course, animal model, DIP dose, and DVG architecture likely affect variations in DVG persistence and association between time of delivery and protection against challenge viruses. Persistence of DVGs in the same physiological space as the infecting virus is undoubtably a key determinant of treatment success.

Two mechanisms of protection have been proposed for DIPs: non-specific immune stimulation or direct interference. DVG-mediated direct inhibition stems from the accumulation of shorter DVGs over full-length STV genomes, resulting in depletion of viral and cell-based components vital for a productive viral lifecycle.^8^ Therefore, direct interference mechanisms would necessitate accurate recognition of promoter regions by the viral replication complex to confer DIP inhibitory efficacy. Promoters regulating paramyxovirus genomic replication are in the leader and trailer regions of the genome. In HNVs, these regions are highly conserved, with only 2 nt differences in the leader sequence between NiV-M and NiV-B, and 11 and 3 nt differences in the leader and trailer sequences, respectively, between NiV-M and HeV. In vitro data suggest that the viral replication complexes of HNVs can recognise each other’s replication and transcription promotors.^31^, ^32^, ^33^ We found that NiV-M-derived DIPs mediated NiV-B inhibition in vitro and a single prophylactic NiV-M-derived DIP treatment given 3 days prior to NiV-B challenge in hamsters resulted in increased chances of both survival and asymptomatic infection. These data indicate that the NiV-B proteins involved in replication can utilise the NiV-M promoters contained in the DVGs, and amplify their numbers to concentrations capable of inhibiting NiV-B replication.

Against the more distantly related HeV, however, DIP treatment was largely ineffective, indicating that non-specific immune stimulation was either absent or insufficient to confer protection. Non-specific immune stimulation has been demonstrated for DIPs against other viruses. For example, influenza-derived DIPs with distinct architectures have been shown to be highly effective against strains different from the strain of DVG origin^13^^,^^23^^,^^34^, ^35^, ^36^ and to induce non-specific protection, with IAV DIPs demonstrating in vitro inhibition against SARS-CoV-2, respiratory syncytial virus, yellow fever virus, and ZIKV,^37^^,^^38^ and in vivo protection from influenza B virus and pneumonia virus of mice.^30^^,^^39^ Furthermore, myxovirus resistance 1-deficient mice could not be protected by IAV DIP treatments,^40^ suggesting that the interferon response is critical to the antiviral efficacy of IAV DIPs. In both these and our previous studies, non-specific immune stimulation was evaluated as a putative contributor to protection. However, we previously showed that NiV DIPs elicit only transient non-specific immune responses in hamsters, detectable at 1 day and largely absent by day 4,^19^ with similar in vitro findings reported for ZIKV DIPs.^28^ These results, together with the findings presented here, further support direct interference as the predominant mechanism of protection for NiV-derived DIPs.

Several factors may explain the reduced efficacy of NiV-M-derived DIP treatment against HeV: (1) relatively reduced replication of DVGs by the HeV replication machinery due to less efficient promoter recognition; and/or (2) the more rapid onset of acute clinical disease in this stringent disease model, which may limit the window for DIP inhibition to provide meaningful clinical benefit. For the former, although in vitro data shows that HNV promotor recognition is possible for all tested species, this is under very specific and controlled conditions during minigenome assays^31^, ^32^, ^33^ which may not accurately recapitulate the cellular environment faced by the virus and DVGs during and active infection, both in vitro and in vivo. Minor variances in recognition and utilisation of promoter sequences may lead to less DVG replication and therefore less overall inhibitory pressure on viral replication. This, coupled with a more acute and severe disease course seen for HeV, and the absence of non-specific DIP-mediated protection, may account for the failure in protection observed.

While efficacy may vary in part be due to inherent differences in disease course and clinical presentations between the HNVs in this model, overall, greater genetic distance from the NiV DVG origin resulted in poorer prophylactic efficiency. As direct interference appears critical to HNV DIP treatment, their broad clinical applicability will rely on the feasibility of rapidly developing DIPs for new HNV strains or species. The DIPs described here and in our previous studies were designed using the well-established NiV-M reverse genetic system, a system integral for DVG generation and DIP production.^18^ Reverse genetic systems for both NiV-B and HeV are now also available.^41^, ^42^, ^43^ Although we have not yet undertaken efforts to develop DIPs for these viruses, these systems could, in principle, be used to generate NiV-B- and HeV-based DIPs using the same methods previously applied for NiV-M. Furthermore, future studies may explore the utility of combination DIP therapy to enhance efficacy against emerging henipaviruses.

Potential strategies to enhance efficacy include more frequent dosing and optimised delivery methods. Because cellular DVG amounts appear to impact efficacy, repeated treatment may be considered in certain clinical or exposure scenarios. Importantly, we and others have demonstrated that DIP treatment is seemingly benign, with no evidence of lung pathology observed, nor abnormal markers of inflammation or cytokine activation detected post-delivery.^10^^,^^11^^,^^14^^,^^16^^,^^19^^,^^23^^,^^27^ Additionally, in these studies, no clinical signs were observed in hamsters treated prophylactically for up to 7 days or monitored for up to 14 days to assess DIP persistence. Taken together, repeated doses would likely be safe and well tolerated if required. Furthermore, here we delivered DIPs intranasally dropwise with a pipette. While hamsters are closely monitored post treatment to ensure no treatment volume is expelled (via sneezing, head shaking, or leaking via gravity), ensuring that a standard dose is given to every animal every time can be challenging. We believe that mucosal delivery is ideal for DIP treatments targeting respiratory infections, as the treatment route should ideally match the presumed natural route of HNV infection, and self-administration for patients would be easy and painless. Nasal delivery devices such as nasal spray pumps and pressurised metered-dose inhalers could be investigated to assess their potential practicality for enhancing DIP efficacy.

This study has some limitations to note. Firstly, we evaluated only a subset of DIP candidates; others might produce different or more potent non-specific effects. Secondly, delivery via intranasal pipette may have resulted in variability in dosing and thus variation in outcomes. While our study focused on intranasal administration, other routes or dosing regimens could influence efficacy and safety profiles, and further work is needed to optimise delivery parameters. Thirdly, we did not assess longer-term outcomes beyond the acute disease phase, including potential late sequelae or viral persistence. Finally, although our findings demonstrate promising efficacy in hamsters, extrapolation to humans remains uncertain due to differences in host biology and disease progression.

The public health threat of HNV infections remains considerable, as NiV is a highly lethal disease with outbreak potential in densely populated regions, and novel henipaviruses with unknown clinical significance continue to be identified.^44^, ^45^, ^46^ Therapeutic candidates that can be rapidly developed, are safe and easily administered, and confer robust pre-exposure protection would be crucial tools during outbreak responses and for pandemic preparedness. A DIP-based treatment approach, similar to that described here, offers several benefits for use as a medical countermeasure, with multiple studies confirming both safety and efficacy of DIP treatment for a wide range of viral pathogens. Overall, these data support direct interference-mediated protection by NiV-derived DIPs and demonstrate the efficacy of DIPs in pre-clinical models justifying continued studies examining the utility of DIP-based treatments and therapies for henipaviruses and other pathogenic viruses.

## Contributors

Conceptualisation–SRW, JRS, STN, CFS; Data curation–SRW, JRS; Formal analysis–SRW, JRS, KAD; Funding acquisition–SRW, JRS, JMM, STN, CFS; Investigation–SRW, JRS, JRH, JDC, SCG, KAD, TES, FEMS, SER; Methodology–SRW, JRS; Project administration–SRW, JRS, CFS; Supervision–JRS, JMM, STN, CFS; Validation–SRW, JRS; Verification of underlying data—SRW, JRS, KAD; Visualization—SRW; Writing—original draft–SRW, JRS; Writing—review & editing–SRW, JRS, KAD, TES, STN, CFS. SRW and JRS accessed and verified the data. All authors read and approved the final version of the manuscript.

## Data sharing statement

Data are available upon reasonable request.

## Declaration of interests

Authors have no conflicts of interest to report.
